# *In vitro* study of a new theranostic smart niosomal nanostructure for direct delivery of docetaxel via anti-PSMA aptamer

**DOI:** 10.1016/j.heliyon.2024.e37341

**Published:** 2024-09-02

**Authors:** Negar Nasri, Shaghayegh Saharkhiz, Ghasem Dini, Fariba Ghasemvand

**Affiliations:** a Department of Biotechnology, Faculty of Biological Science and Technology, University of Isfahan, Isfahan, 81746-73441, Iran; b Department of Nanotechnology, Faculty of Chemistry, University of Isfahan, Isfahan, 81746-73441, Iran; c Center for Advanced Technologies, Adam Mickiewicz University, Uniwersytetu Poznańskiego 10, 61-614, Poznań, Poland

**Keywords:** Aptamer-conjugated, Prostate cancer, Anti-PSMA aptamer A10, Niosome, pH-sensitive, PSMA-Positive, Docetaxel, Theranostics

## Abstract

In this study, a novel quantum dot (QD)-labeled specific anti-prostate-specific membrane antigen (PSMA) aptamer sequence was conjugated to a pH-responsive niosomal particle platform for delivery of docetaxel (DTX) components. The target cells were overexpressed PSMA. This strategy can minimize the systemic toxicity prevalent in DTX. Synthesis of pH-responsive niosomes was achieved by using thin-film hydration. The conjugation of the aptamer A10 to the niosomal particle was done via a disulfide bond. Furthermore, CdSe/ZnS QDs were fabricated using a hot-injection process, then were functionalized with mercapto propanoic acid (MPA) ligands and attached to the 3’ terminal of aptamer via an Amide bind. Moreover, several characterization analyses including dynamic light scattering (DLS), zeta potential, Fourier transform infrared spectroscopy (FTIR), X-ray diffraction (XRD), scanning electron microscope (SEM), and transmission electron microscope (TEM) were performed. Additionally, 3-(4,5-Dimethylthiazol-2-yl)-2,5-Diphenyltetrazolium Bromide (MTT) and apoptosis assays, as well as fluorescence microscopy, were used to assess the performance of the fabricated system. The data revealed a homogenous round-shaped population of niosomes with an average size of 200 nm and a negative surface charge was synthesized successfully. The FTIR and XRD evaluations confirmed the fabrication of both QDs and niosomes and the bioconjugation processes. The drug release happened in a controlled manner with a pH-sensitivity feature. The cellular uptake of aptamer-conjugated particles enhanced and consequently caused more cytotoxicity of prostate cancer cells with overexpression of PSMA. Furthermore, the QDs provided an ability to trace the treatment and its uptake via the targeted tissue. Overall, this study contributed to the development of a low-risk, highly specific platform for the delivery of both therapeutics and imaging agents.

## Graphical abstract

**Figure:**
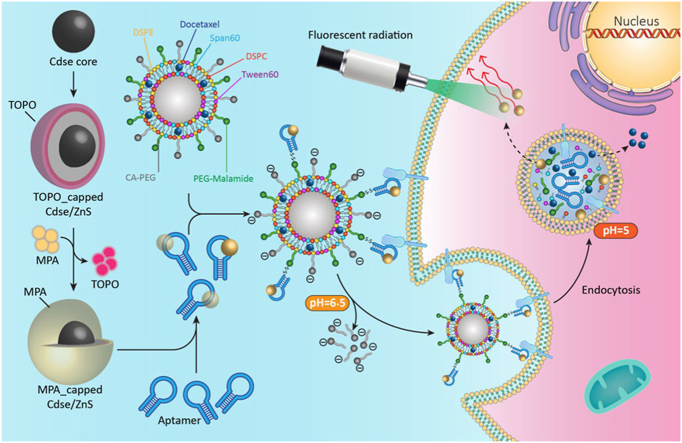

## 1 Introduction

According to previous studies worldwide, prostate cancer (PCa) is the second most common malignancy after lung cancer and the fifth cause of death in men. Based on the Global Cancer Observatory (GLOBOCAN) estimated in 2018, there were 1,276,106 new cases of PCa recorded, accounting for 7.1 % of all cancers in men and causing 3.8 % of all deaths (358,989) in men [[1], [2], [3]]. More than 80 % of advanced PCas respond well to androgen deprivation treatment [4]. Among the treatments that were considered for prostate cancer was hormonal treatment. Sex hormone signaling is regulated in the hypothalamus-pituitary-gonadal axis. The strategy of hormonal therapies is to disrupt the hypothalamus-gonadal axis to stop testosterone production. Synthetic versions of estrogen such as diethylstilbestrol suppress the testosterone level in the serum by increasing the negative feedback in the hypothalamus. However, this method was limited due to side effects such as gynecomastia, sexual dysfunction, and venous thromboembolism [5]. Approximately half of the advanced PCa patients become resistant to treatment after several years and require other palliative methods such as estramustine phosphate (EMP) and steroids. The outcomes are disappointing since survival was not increased [6]. Treatment for PCa commonly involves chemotherapy, but only after it has advanced to the stage of castration-resistant prostate cancer (CRPC). Taxanes family including Paclitaxel (PTX) and Docetaxel (DTX) typically affects cell division by interfering with the normal function of microtubule growth and leading to programmed cell death or apoptosis in cancer cells [7]. DTX inhibits microtubules, causing cell death in the G2/M phase of the cell cycle, approved by the FDA as the mainstay treatment against HRPC.

The only commercial formulation of DTX containing polysorbate 80 is Taxotere [8]. Owing to multidrug resistance, DTX and its excipients possess severe toxic side effects as well as being highly lipophilic and practically insoluble in water. Unfortunately, the therapeutic benefit of DTX is limited by side effects specifically dose-limiting myelosuppression. Due to this unfortunate situation, it is critical to develop novel combination therapies or delivery systems to enhance its anti-tumor efficacy and reduce its side effects [9].

Recent developments in nanotechnology and medicine have garnered considerable attention thanks to the interacting ability of nanostructures with the body at a molecular level. Researchers have developed novel cancer therapies that use nanomedicine to enhance drug specificity and efficacy, resulting in maximum effectiveness and minimum side effects. To illustrate, polymeric micelles, mesoporous silica particles, dendrimers, liposomes, and niosomes have been utilized to transport various therapeutics agents such as antiangiogenic agents, chemosensitizers, cytotoxic drugs, and small interference RNAs [[10], [11], [12]]. Vesicular systems, in particular niosomes, are widely considered drug carriers [13]. Niosome is formed by nonionic surfactants through self-assembly in aqueous solutions when agitated by an ultrasonication device or heated. In niosomes, phospholipids have been replaced with nonionic surfactants in the membrane-forming constituents to overcome the liposome-related disadvantages, including lack of chemical stability, the tendency of phospholipids to oxidize, high production costs, special handling, and storage requirements. Hydrophobic and hydrophilic drugs can be simultaneously encapsulated in the lipid system. Hydrophobic drugs can be loaded between the two layers and hydrophilic drugs can be loaded in the center of the system [14]. In addition, niosomes demonstrate osmotically active properties, non-toxic and non-immunogenic nature, biocompatibility, and biodegradability. It has been shown that niosomes can deliver drugs using multiple delivery routes, including oral, parenteral, dermal, transdermal, ocular, and pulmonary. Targeted drug delivery, particularly in cancer therapy, relies heavily on the functionalization of niosomes. Today, with the help of nanotechnology, drug delivery systems have been able to acquire a range of new features such as delivery of poorly soluble drugs, increased cell permeability, improved transmembrane delivery, co-delivery of multiple therapeutic agents with different properties, and site-specific targeting of delivery systems. Nanocarriers can be targeted passively as well as actively within tumor cells to prevent their accumulation. Passive targeting occurs in the bloodstream as a result of two physiological processes: convection and diffusion. In tumor vascular endothelium, large molecules are transported by convection through an osmotic pressure gradient in blood flow. Concentration gradients allow low molecular weight and highly lipophilic compounds to diffuse across the cell membrane via diffusion. Due to the enhanced permeability and retention (EPR) effect, nanocarriers can accumulate in tumors [15]. To produce pH-responsive polymers, charge-reversible linkers from niosomal formulations like citraconic anhydride (CA) are effective elements to release drugs in acidic pH conditions of cancer cells. In acidic conditions, the bond cleavage causes a positive charge to appear on the surface of a citraconic-amide bond formed by an amine functional group and CA [16]. In active targeting, surfaces of nanocarriers are modified to bind to overexpressed receptors in tumor cells, causing a specific interaction between target cells and carriers. Nevertheless, first, nanocarriers must reach the tumor cells and second, the EPR effect must still be utilized to bind the target cells [17].

A large amount of attention is paid to biomarkers when it comes to active drug delivery. To achieve this, the desired drug should be delivered precisely to cells that express specific biomarkers, such as the ones in diseased cells. There are three mechanisms of biomarker expression in tumor cells: i) mutations that lead to structural differences, ii) overexpression, which may be helpful for tumor progression, and iii) post-translational modifications, altering the structural-functional properties [18]. Thus, smart molecules are required to distinguish between biomarkers. In addition to their molecular recognition capabilities, aptamers are very specific and can be differentiated between a wide range of molecules, leading to broad usage in targeting intracellular, extracellular, and cell surface tumor markers. In tumor cells, aptamers can deliver drugs, macromolecules, or nanocarriers. Owing to receptor-mediated endocytosis, cellular internalization can be increased by specific interaction with surface receptors [19]. As a result, RNA and DNA aptamers were successfully selected to interact with potential cancer cell surface receptors. Among the notable examples of fluorinated RNA aptamers are A10 and A9, which are designed to target purified prostate-specific membrane antigens (PSMA). It is an integral membrane glycoprotein of type II which plays an attractive role in diagnosing and treating PCa. In normal cells in prostate tissue, the expression level of PSMA is low, but only 1 % to one-thousandth of it can be expressed in PCa tissue, indicating the high specificity. Furthermore, PSMA expression is independent of androgen levels, which remain high throughout the disease. After endocrine therapy, hormone-refractory PCa is crucial for these patients. A9 and A10 are the first generation of classic aptamers used in this method [17]. As the target protein, these aptamers bind to PSMA and ingest it [20].

**Scheme 1:**
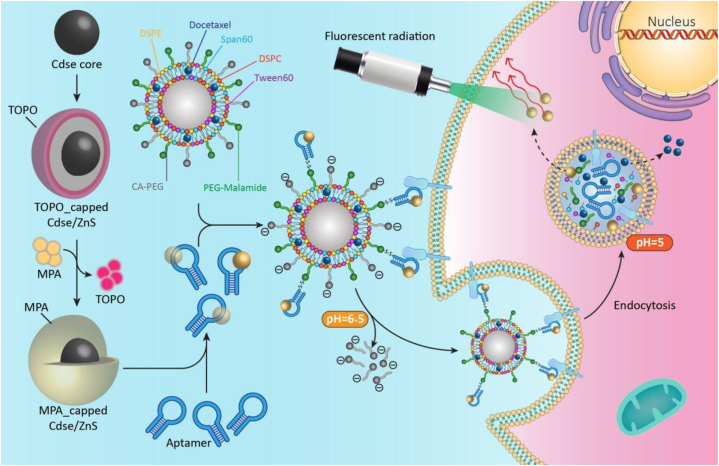
The overall strategy, fabrication, and assessment methods were used in this study, (CdSe core = Cadmium-Selenide core, TOPO = Trioctylphosphine oxide, MPA = Mercaptopropionic Acid, CdSe/ZnS = Cadmium-Selenide/Zinc-Sulfide core/shell Quantum dots, DSPE = 1,2-Distearoyl-sn-glycero-3-phosphorylethanolamine, CA-PEG = Citraconic Anhydride-Polyethylenglycol, PEG-Maleimide = Polyethylenglycol-Maleimide, DSPC = Distearoylphosphatidylcholine).

In this work (Scheme 1), firstly, CdSe/ZnS quantum dots (QDs) were synthesized and characterized and then attached to a specific anti-PSMA aptamer A10 via an amide bond. Secondly, the QD-labeled aptamer was bioconjugated to a fabricated pH-sensitive niosomal system for targeted delivery of DTX to the prostate cancer cell line. The theranostic effect of the fabricated platform was assessed on both PSMA-positive LNCap and PSMA-negative L929 cell lines. The biological activity of the particles was evaluated using the MTT method. The amount of apoptosis was also measured by apoptosis kit and flow cytometry. In addition, the CdSe/ZnS QDs were used to track and prove the successful performance of the smart system via bioimaging.

## 2 Material and methods

### 2.1 Materials

Previously, 1,2-Distearoyl-sn-glycero-3-phosphorylethanolamine-Citraconic Anhydride-Polyethylenglycol2000 (DSPE-CA-PEG2000) was prepared by our team [16]. Sorbitan stearate 60 (Span 60, ≥99 %), polyethylene glycol sorbitan monostearate 60 (Tween 60, ≥99 %), 1,2 stearoyl-sn-glycerol-3-phosphoethanolamine-N-[maleimide (polyethyleneglycol)-2000] (DSPE-PEG2000-Mal, ≥99 %), 1,2-distearoyl-sn-glycero-3-phosphocholine (DSPC, ≥99 %), polyethylene glycol sorbitan monolaurate (Tween 20, ≥99 %), cholesterol (≥99 %), hydrochloric acid (HCl, ≥37 %), ethanol-1,1,2,2-d4-amine (≥99 %), bovine serum albumin (BSA, ≥98 %), Roswell Park Memorial Institute-1640 medium (RPMI-1640) including 10 % fetal bovine serum (FBS), thiazolyl blue tetrazolium bromide (MTT) powder, Dimethyl sulfoxide (DMSO),threo-1,4-Dimercapto-2,3-butanediol (DTT, ≥99 %), trioctylphosphine (TOP, ≥99 %), trioctylphosphinoxid (TOPO, ≥99 %), zinc dichlorodiphenyltrichloroethane (ZnDDT, ≥99 %) and dimethylsulfoxide (DMSO), chloroform, toluene and diethyl ether (≥ 99.9 %) were purchased from Merck company, Germany. In addition, 2-propanol, ethanol, sodium silicate, Trypsin/EDTA (0.5 %), and penicillin/streptomycin antibiotics were acquired from Thermofisher. Docetaxel (≥99 %) was purchased from Actore Co, Iran. Also, cadmium oxide (CdO, ≥99 %), octadecane (CH3(CH2)16CH3, ≥99 %), oleic acid (cis-9-Octadecenoic acid, ≥90 %), selenium black (Se, 99.99 %), N-Ethyl-N′-(3-dimethylaminopropyl)carbodiimide polymer-bound (EDC), 1-Hydroxy-2,5-pyrrolidinedione (NHS, ≥98 %) and mercaptopropionic acid (MPA, ≥99 %) were purchased from Sigma-Aldrich, Germany. Besides this, thiolated RNA aptamer A10 (Apt A10) strand oligonucleotides purified by high-performance liquid chromatography (HPLC) were purchased from Copenhagen, Denmark Biological Engineering Technology & Services and suspended in 100 mM DTT buffer. The relevant DNA sequence for PSMA detection was as follows [21]:

(5′-thiol GGGAGGACGAUGCGGAUCAGCCAUGUUUACGUCACUCCUUGUCAAUCCUCAUCGGC–NH_2_ 3′)

### 2.2 Methods

#### 2.2.1 Synthesis of CdSe/ZnS QDs

The first step of synthesizing CdSe cores included mixing 205.45 mg of cadmium oxide with 2.5 mL of oleic acid and 2.5 mL of octadecane in a balloon under nitrogen gas and 120 °C. In the next step, the temperature was raised to 280 °C under a magnetic stirrer, and 15.8 mg of selenium black was dissolved in 350 μL of TOP and then added to the above solution and heated for 30 min at 280 °C. A second step involves synthesizing the shell of QDs by decreasing the system's temperature to 225 °C and adding 34.6 mg of ZnDDT to 350 μL of TOP solution then adding to the above solution. In the meantime, 87.5 mg of TOPO was added. We allowed the solution to remain at 240 °C, then raised the temperature to 280 °C and shut the system down after 20 min. A centrifuge of 10,000 rpm was performed for 15 min, followed by a wash with 30 mL of toluene. In the refrigerator, we stored the remaining sediment in 20 mL of toluene and 0.830 g of TOPO [22].

#### 2.2.2 Synthesize of drug-loaded pH-responsive niosome

pH-responsive niosomes (pHSNs) were fabricated using a thin-film hydration method [16]. For this purpose, previously prepared DSPE-CA-PEG2000 according to Ref. [16], In this experiment, as niosome components, DSPE-CA-PEG2000, DSPE-PEG2000-Mal, DSPC, Tween 60, Span 60, and cholesterol were dissolved in 5 mL of chloroform and stirred for 15 min in a molar ratio of 5:1.5:3.5:15:45:30. After adding 2 mg of DTX to the mixture, a thin film was formed by evaporating the mixture under vacuum at 60 °C using a rotary evaporator. Following this, 10 mL of preheated buffer phosphate saline solution (PBS, pH 7.4, 60 °C) was added and stirred for 1 h at 60 °C for the preparation of multi-lamellar niosomes. A probe sonicator was used to sonicate the solution for 1 h in an ice bath to reduce the size of the particles and form the single-lamellar cationic niosomes. As the last step, purification was achieved using dialysis (24 h, 12 kDa dialysis bag, against PBS) [23].

#### 2.2.3 Ligand exchange of CdSe/ZnS QDs

CdSe/ZnS Qds capped with TOPO were dispersed in PBS by replacing TOPO with mercaptopropionic acid (MPA). In other words, carboxylate ligands of MPA molecules provide water dispersibility to QDs. A typical concentration of 5 μL of MPA and 1 mL of PBS buffer was poured into 1 mL of dispersion containing CdSe/ZnS QDs and stirred overnight. Several times of centrifugation were performed on QDs to remove the MPA residue [24].

#### 2.2.4 Bioconjugation of QDs with aptamer

EDC, NHS, and QDs reacted in dark conditions for 2 h at 4 °C, with molar ratios of 1000:1000:1. The functionalized QDs were ultrafiltered (30k MW cut-off) and hybridized with RNA-aptamer (2 mL, 100 mM) in PBS for 3 h. Conjugated QDs with RNA were purified by ultrafiltration (30k Mw cut-off) [25].

#### 2.2.5 Conjugation of A10 aptamer to the surface of pHSNs

After connecting the end (3′) of the aptamer to QDs, to connect the aptamer to the surfaces of niosomes, the thiol end (5’) of the aptamer was activated by the following process. In order to functionalize pHSNs with Apt10, we used thiol-maleimide cross-linking to create an S-S bond. To conjugate C6-thiolated Apt10 in nuclease-free water, 100 mM DTT (pH 8.3–8.5) was used to deprotect Apt10-SH for 30 min at room temperature in preparation for conjugation. Apt10-SH is then purified by precipitation using 3 vol of absolute ethanol and 0.1 vol of sodium acetate 3M. Re-suspension of precipitated Apt10-SH in binding buffer (2.5 mM MgCl2, 1× PBS, pH 7.4), heating for 10 min at 70 °C, and cooling rapidly on ice was carried out. The resulting deprotected Apt10 was conjugated to the thiol group of DSPE-PEG2000-Mal, which was present in the structure of pHSNs, at a 0.5:1 M ratio by incubation overnight at 4 °C followed by dialysis to remove free Apt10 from the Apt10-niosome suspension [26].

### 2.3 Characterizations

Dynamic light scattering (DLS) and zeta potential (HORIBA, Scientific SZ-100, Japan) were used to evaluate the size and surface charge of QDs before and after ligand modification with MPA. Also, to prove the exact size of the QDs, a transmission microscope (HT7800, Hitachi, Japan) was used. In addition, to evaluate the successful synthesis of CdSe/ZnS core/shell QDs, X-ray diffraction (XRD, D8 Advance, Bruker, Germany) analysis was used. Furthermore, the Fourier transform infrared spectroscopy (FTIR, JASCO, Japan) technique was performed to evaluate the correct construction of CdSe/ZnS QDs and MPA-QDs. To estimate the emission and excitation wavelength of QDs, fluorescence spectroscopy was used between 200 and 800 nm. Additionally, dynamic light scattering (DLS) and zeta potential measurements were conducted to assess the size and surface charge of the pH-responsive niosomes both before and after drug encapsulation and surface modification. FTIR analysis was used to verify the successful construction of the empty niosomes, those encapsulated with the drug, and the niosomes with the A10 aptamer attached to their surface. Moreover, scanning electron microscopy (SEM, Leo 1430 VP, Germany) was employed to examine the morphology of the niosomes before and after modification.

### 2.4 Bio-activity assessments

#### 2.4.1 *In vitro* entrapment efficiency assessment

To measure niosomal drug content, 300 μL of a drug-loaded suspension was mixed with 2700 μL of 2-propanol (1 % w/v in water) and stirred for 48 h at room temperature. To separate niosomal fragments from the released drugs, the supernatant was centrifuged at 10,000 rpm and analyzed by high-performance liquid chromatography (HPLC, LC-20A, Shimadzu, Japan). To carry out chromatographic separations, the Unitary C18 column (250 mm × 4.6 mm) was used. The mobile phase consisted of acetonitrile and water (60/40, v/v), and the detection wavelength was 230 nm [27]. To determine the DTX concentration, a calibration curve of the standard DTX solution was used; the curve was linear between 0.5 and 250 μg/mL with a correlation coefficient of 0.9998. Then, the percentage of entrapment efficiency (EE%) was calculated using equation (1) [24]:

(1) $$\text{Entrapment}\;\text{efficiency}\;\left(\%\right)=\frac{\text{Mass}\;\left(\text{total}\;\text{drug}\;–\;\text{unloaded}\;\text{drug}\right)\;}{\;\text{Mass}\;\left(\text{total}\;\text{drug}\right)\;}\times 100$$

#### 2.4.2 Stability evaluation

In order to determine whether the samples are stable over time, DLS analysis was repeated after 4 months of storage at 4 °C to monitor their size changes, as well as their EE%, which were measured again to investigate the capability of the particles to keep their components by passing the time.

#### 2.4.3 MTT assay

In this study, cell viability of different concentrations of pH-responsive niosome suspensions was evaluated using the MTT assay on both LNCap and L929 cell lines purchased from Pasteur Institute Cell Bank of Iran, Tehran. In brief, 10 ^4^ cells were seeded into wells of 96-well plates, filled with 200 μL RPMI-1640 cell culture medium, and incubated at 37 °C and 5 % CO2 for 24 h. Afterward, each well was refilled with 200 μL of fresh medium containing drug-loaded niosomes (with and without surface modification) as well as empty aptamer conjugated niosomes, pure DTX, and pure aptamer A10. At this point, the cells were incubated for 24 and 48 h at 37 °C. After two washes with PBS, the cells were exposed to 200 μL of culture medium containing MTT (5 mg/mL in PBS), and incubated at 37 °C for 2 h. After an hour of incubation, the wells were declined and replaced with 200 μL of DMSO, and their absorbance was determined with an ELISA reader (Bio-Rad, Hercules, USA).

#### 2.4.4 Apoptosis investigation

To detect apoptosis, we cultured 5 × 10 ^5^ LNCap cells to approximately 70 % confluency in 10 mm dishes and assessed the percentage of apoptotic cells using flow cytometry. Each dish's cells were treated with 1 mL of fresh medium containing either empty niosomes, drug-loaded niosomes, aptamer-niosomes, DTX, or a control. After 24 h of treatment, we added 5 μL each of Annexin V and Propidium iodide (PI) dyes to the cells and incubated them in the dark for 15 min. The cells were then analyzed using flow cytometry (C6, BD Accuri, USA) [28].

#### 2.4.5 Bioimaging

To evaluate and prove that the system is theranostic, a fluorescence microscope was used. LNCap cells were incubated with aptamer-conjugated niosomes for 24 h at 37 °C with 5 % CO2. Post-incubation, the cell nuclei were stained and fixed using 1 mg/mL of 4′,6-diamidino-2-phenylindole (DAPI) dye in methanol, followed by a 5-min incubation in a dark room. The cells were then analyzed using an OLYMPUS BX61 fluorescence microscope (Japan).

#### 2.4.6 Statistical analysis

The evaluation of the significance of data was done using SPSS software (IBM, version 21, parametric analysis of variance, ANOVA (Tukey)) and the determination of the outcomes was performed based on the *P* ≤ 0.001 for release tests, and *P* ≤ 0.05 for MTT assay.

## 3 Results and discussion

### 3.1 Characterization of QDs

To evaluate the size and surface charge of CdSe/ZnS and CdSe/ZnS-MPA QDs, DLS analysis was performed. According to the results, the size of CdSe/ZnS and CdSe/ZnS-MPA QDs were 7.37 ± 0.11 and 8.4 ± 0.21 nm with a polydispersity index (PDI) of 0.026 ± 0.005 and 0.022 ± 0.002, respectively. The achieved PDI values were the indication of the quality and monodispersity of the QDs [29]. Additionally, the surface charge of CdSe/ZnS QDs was approximately 0.8 ± 0.01 mV. However, when the TOPO surface ligand of the QDs was exchanged with MPA, this charge shifted to the more negative amounts of −10.2 ± 0.1 mV, approving the successful exchange process [30].

**Fig. 1:**
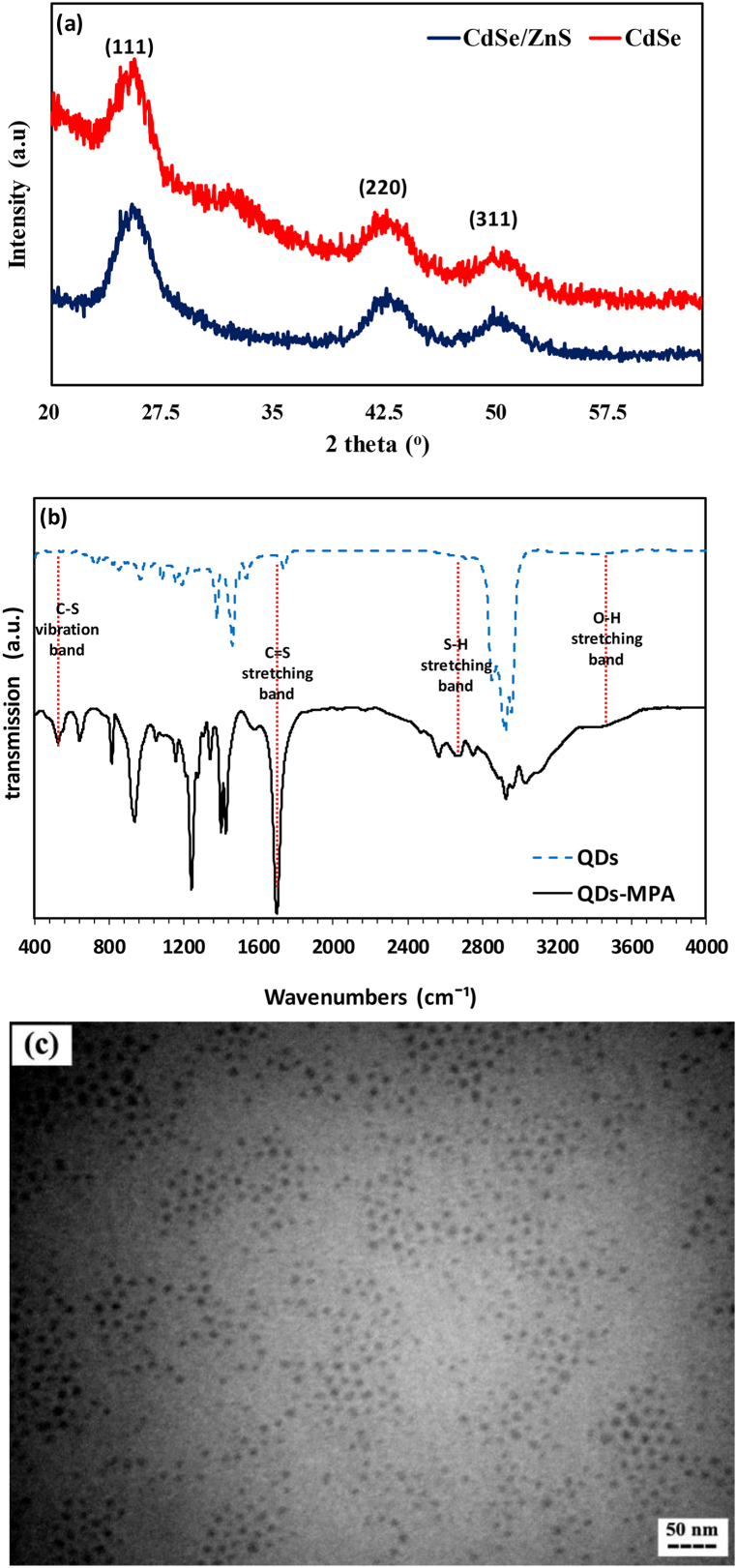
(a) XRD patterns of CdSe core and CdSe/ZnS QDs, (b) FTIR spectra of TOPO-capped and MPA-capped CdSe/ZnS QDs, and (c) TEM image of CdSe/ZnS QDs.

XRD analysis was also performed to investigate the crystallinity and phase composition of fabricated QDs (Fig. 1a). Based on the achieved pattern of CdSe/ZnS QDs, three diffraction planes of (111), (220), and (311) emerged at three distinct 2θ of 25.8, 42.95, and 50.35°, attributing to the cubic structure of ZnS (ICDD PDF no. 00-019-0191). In addition, concerning the XRD pattern of the CdSe core, the same (111), (220), and (311) planes were observed at 25.5, 42.65, and 50.75°, resembling the cubic structure of CdSe structure (ICDD PDF no. 01-080-0020) [16,31].

To evaluate the TOPO ligand exchange with MPA, FTIR analyses were done on both CdSe/ZnS and CdSe/ZnS-MPA QDs (Fig. 1b). The results revealed emerging of three distinct bonds at 527.13, 2668.99, and 3453.88 cm^−1^ in the spectrum of the CdSe/ZnS-MPA, while they were absent at the spectrum of TOPO capped CdSe/ZnS QDs. These bonds were attributed to the C-S vibration bond, S-H stretching bond, and O-H stretching bond, respectively, representing the successful replacement of TOPO ligands with MPA on the surface of QDs [32]. Additionally, in both spectra, a prominent bond was seen at 1704.76 cm^−1^ due to the C=O stretch bond of protonated carboxylic acid [33]. However, the intensity of the C=O bond was higher at MPA-capped QDs than that for TOPO-capped ones due to the R-COO^-^ groups of MPA.

Moreover, the morphology of the QDs was investigated using TEM (Fig. 1c). According to the obtained image, the size of the QDs was estimated at around 7 nm which was in line with the DLS results. Also, their distribution was homogenous and confirmed the observed PDI in the previous analysis.

**Fig. 2:**
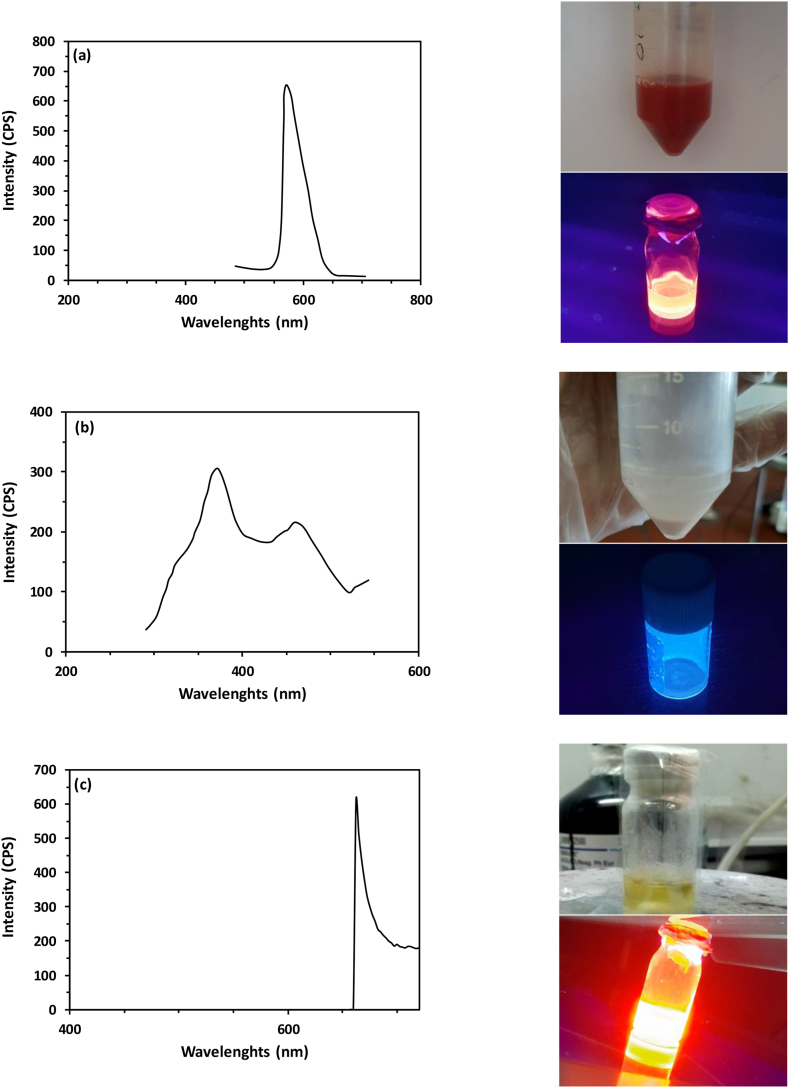
Fluorescence spectroscopy of (a) bare CdSe/ZnS QDs, (b) MPA-capped CdSe/ZnS QDs, and (c) AptA10-conjugated CdSe/ZnS QDs.

The emission peaks of TOPO-capped QDs, MPA-capped QDs, and AptA10-conjugated QDs were determined using a fluorescence spectrometer. The outcomes revealed an emission peak of 571.7 nm for TOPO-capped CdSe/ZnS QDs which presented an orange color (Fig. 2a). After the ligand exchange of TOPO with MPA, a blueshift was observed and CdSe/ZnS-MPA QDs showed an emission maximum peak at 375.8 nm with a blue color (Fig. 2b). Additionally, after the conjugation of MPA-capped QDs with the A10 aptamer strand, their emission peak shifted to 662.5 nm, emitting a red light (Fig. 2c). Overall, these fluorescence spectra changes can be an approval of the successful process of surface modifications of QDs [34,35].

### 3.2 Characterization of niosome nanoparticles

The evaluation of the size and surface charge of synthesized nano-niosomes was done using DLS and zeta potential analyses. Table 1 presents the obtained size and zeta potential of empty pH-sensitive niosomes (pHSNs), DTX-loaded pH-sensitive niosomes (DTX-pHSNs), and AptA10-DTX-loaded pH-sensitive niosomes (AptA10-DTX-pHSNs). According to the results, the size of pHSNs was 173.3 ± 1.4 nm with a neutral surface charge of 1.2 ± 0.12 mV. This neutral surface charge can be proof of DSPE-CA-PEG2000 incorporation into the niosomal structure [16]. Encapsulation of DTX between the bilayer of the pHSNs did not cause the negotiable size and surface charge changes, presenting a size of 181.5 ± 2.6 nm with a −1.8 ± 0.2 mV of surface charge. However, the attachment of AptA10 strands to the surface of the DTX-pHSNs significantly led to an approximately 40 nm increment in size, revealing a size of 220.1 ± 3.1 nm. Additionally, the surface charge of Apt10-DTX-pHSNs was −16.8 ± 0.9 mV which was due to the presence of nucleotide residues with a negative charge on the surface of the particles, proving the successful conjugation of the aptamers to the surface of the DTX-pHSNs [36]. It is noticeable that the PDI of pHSNs, DTX-pHSNs, and AptA10-DTX-pHSNs were 0.03 ± 0.005, 0.05 ± 0.01, and 0.13 ± 0.03, respectively, confirming the monodispersity of the fabricated particles.

**Table 1:** The size and surface charge of pHSNs before and after drug loading and aptamer conjugation.

Sample	Size (nm)	PDI	Zeta potential (mV)
**Empty-pH-sensitive niosomes (pHSNs)**	173.3 ± 1.4	0.03 ± 0.005	1.2 ± 0.12
**DTX-loaded pH-sensitive niosomes (DTX-pHSNs)**	181.5 ± 2.6	0.05 ± 0.01	−1.8 ± 0.2
**AptA10-DTX-loaded pH-sensitive niosomes (AptA10-DTX-pHSNs)**	220.1 ± 3.1	0.13 ± 0.03	−16.8 ± 0.9

**Fig. 3:**
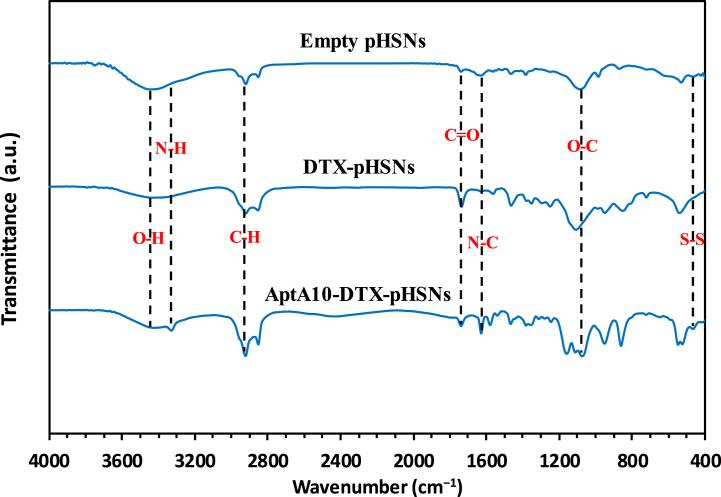
FTIR spectra of empty pHSNs, DTX-pHSNs, and AptA10-DTX-pHSNs.

The evaluation of surface functional groups of the empty pHSNs, DTX-pHSNs, and AptA10-DTX-pHSNs was performed using FTIR analysis (Fig. 3). According to the outcomes, three bonds emerged in the spectra of all samples at 1097.29, 1738.51, and 3462.32 cm^−1^ could be attributed to the stretching C-O ester bond, stretching C=O ether bond, and stretching O-H hydroxyl bond, respectively, which can approve the presence of Span 60, Tween 60, DTX, and cholesterol in the structure of the niosomes [37]. Additionally, an alkyl C-H stretching bond was observed at 2926.44 cm^−1^ in all three spectra [16]. Moreover, a band appeared at 1624.73 cm^−1^ in all three spectra which were related to the N-C stretching bond of amide, attributing to the DSPE-CA-PEG2000. However, this bond was more intense in the spectrum of aptamer-conjugated particles than non-conjugated ones due to the formed N-C bond between the N-terminal of aptamer and carboxyl group of CdSe/ZnS-MPA QDs [16,38,39]. In addition, the stretching S-S bond emanated at 527.15 cm^−1^ in the spectrum of AptA10-pHSNs while it was absent in DTX-pHSNs and pHSNs spectra, confirming the formation of the disulfide bond between DSPE-PEG-Maleimide components and thiol group of aptamer A10 [40]. Furthermore, the N-H bond of DTX came out at 3336.24 cm^−1^ [41]. Overall, these results confirmed the correct fabrication of pHSNs and the successful attachment of aptamer and QDs to the surface of the pHSNs.

**Fig. 4:**
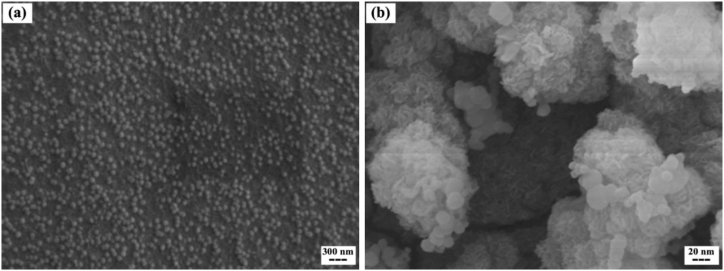
SEM images of (a) empty pHSNs, and (b) AptA10-pHSNs.

In addition, SEM analysis was performed on pHSN and AptA10-pHSN suspensions to investigate their morphology before and after surface modifications. The obtained image of pHSNs presented a homogenous population with approximately 30 nm in size of round-shaped particles (Fig. 4a). Moreover, the presence of aptamer on the surface of the spherical pHSNs is obvious. The size of AptA10-pHSNs was estimated at around 80 nm based on the SEM image (Fig. 4b). In addition, no aggregation was observed in the AptA10-pHSN population, proving the PDI values achieved from DLS analyses. It is worth mentioning that the observed size of particles in SEM images was significantly lower than the DLS results because the obtained size from DLS was the hydrodynamic size which is broader than the actual size of the particles [42].

### 3.3 Bioactivity assessments

#### 3.3.1 Entrapment efficiency and release pattern of DTX

**Fig. 5:**
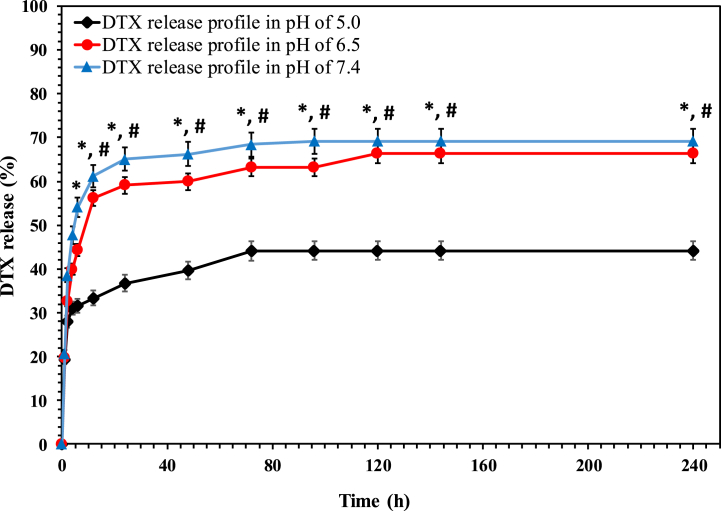
DTX release profile from pHSNs at different pHs of 5.0, 6.5, and 7.4. * and # indicate the significance of pH 5.0 in comparison with pH 7.4 (p ≤ 0.001).

To determine DTX's entrapment efficiency, UV–visible spectroscopy at a wavelength of 220 nm was used. The EE% of hydrophobic DTX in pHSNs was about 81.02 ± 1.9 %. This amount was close to the previous study done by Zarrabi et al. [38]. Additionally, the release pattern of DTX from pHSNs was assessed at two different pHs of 7.4, 6.5, and 5 to simulate the pHs of normal, cancer microenvironments, and lysosome, respectively (Fig. 5). As is obvious, the results revealed that the fabricated pHSN formulation played a significant role in preventing the burst release of DTX molecules in the physiologic pH of 7.4, showing a sustained drug release manner. As can be seen, the decrease in the pH of the medium led to the significant release of DTX and showed a burst release of DTX in the first 4 h due to the breakage of the amide bond between DSPE and CA in acidic conditions, leading to the separation of the CA-PEG2000 layer from the surface of the pHSNs [16]. Furthermore, this pH sensitivity remained in the following hours, owing to the slight pH sensitivity of DSPE nature [38]. Overall, within 240 h of release, about 70 % and 65 % of encapsulated DTX was released at the pH of 5 and 6.5 while this value was approximately half at the pH of 7.4. This type of pH-sensitive release of drugs is efficient for cancer treatment, where the pH of the environment is acidic and different from normal tissues. This release pattern also was in line with the previous pH-sensitive niosomal formulations which were reported, presenting a two-phase drug release in response to the acidic conditions [16,43]. However, the study done by Zarrabi and coworkers on a pH-sensitive liposome for the delivery of hydrophobic curcumin revealed a continuous sustained release manner [38].

#### 3.3.2 Stability of AptA10-DTX-pHSNs

The stability of the fabricated AptA10-DTX-pHSNs was evaluated by measuring the EE% and size of the particles after four months of storage at two temperatures of 4 and 25 °C. The DLS results demonstrated that the size of the particles which were stored at 4 °C only grew up ∼7 nm with a PDI of 0.15 which was ignorable while the stored sample at 25 °C showed approximately 32 nm increase in size with a PDI of 0.21, revealing the sensitivity of sample stability to the temperature during storage. Furthermore, the EE% of DTX also confirmed this sensitivity and showed 8.01 ± 1.2, and 27.5 ± 2.1 % of drug leakage during four months for stored samples at the refrigerator and room temperature (i.e., 20–25 °C), respectively, despite the observation of Akbarzadeh et al. which reported less sensitivity of EE% to the temperature [43].

#### 3.3.3 Cytotoxicity assessment

**Fig. 6:**
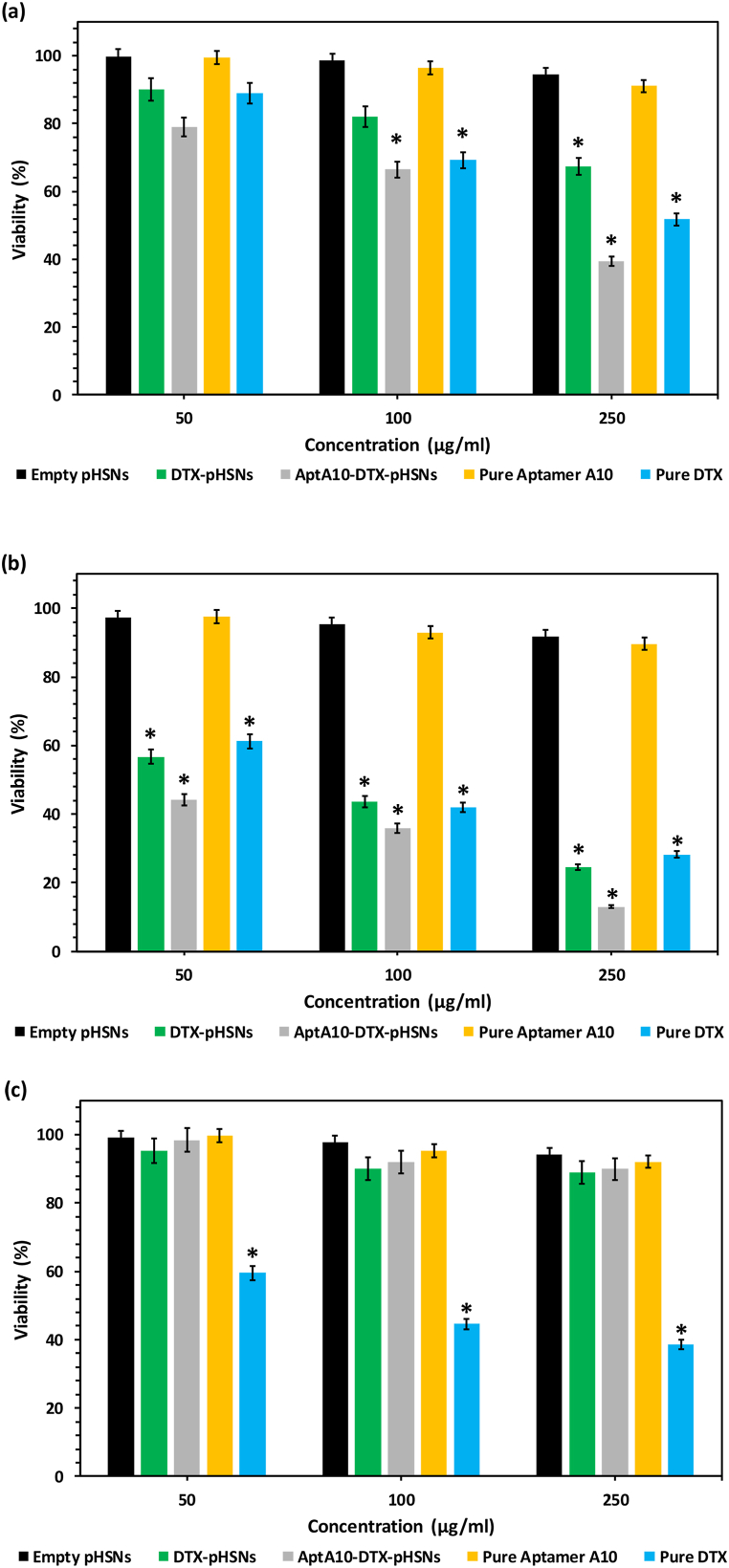
MTT assay results of various treatments on (a) LNCap cell line after 24 h of incubation, (b) after 48 h of incubation, and (c) L929 cells after 48 h of incubation. * is an indicator of the significance of each treatment in comparison with the control group (p ≤ 0.05).

The investigation of cell inhibition performance of various treatments was done using MTT assay on both PSMA-positive LNCap and PSMA-negative L929 cell lines (Fig. 6). The examination was performed at two-time points of 24, and 48h with three concentrations of the treatments (i.e., 50, 100, and 250 μg/mL). The assessments on the LNCap cell line revealed a time and concentration-dependent manner. To illustrate, after 24 h of incubation of LNCap cells with 50, 100, and 250 μg/mL of AptA10-DTX-pHSNs, 79.15, 66.46, and 39.55 % of cell viability was observed, respectively. Then, after 48 h, these values further decreased to 44.17, 35.86, and 12.98 %, respectively (Fig. 6a and 6b). This time-dependent toxicity of the particles could be attributed to the pH sensitivity of the pHSNs because the presence of the buffers in the cell culture media prevented the pH reduction in the first 24 h of incubation, denoting the pH-resistant pattern of the delivery system. Furthermore, the next day, the pH of the media changed to a more acidic pH (the color of the media transformed from pink to yellow) and consequently, the pHSNs responded to the low pH of the environment, causing more drug release and cell growth inhibition [16,44]. In addition, the targeted aptamer-conjugated particles showed significantly better performance in comparison to the non-conjugated particles and pure drugs. For more details, the maximum concentration of AptA10-DTX-pHSNs caused approximately 12, and 16 % more cell deaths than DTX-pHSNs and pure DTX at the time point of 48 h, respectively, confirming the success of the targeting strategy. Additionally, the cellular effects of pure aptamer and empty pHSNs on PSMA-positive cells were negligible. Moreover, the MTT assay results of three concentrations of the same treatments on the L929 cell line did not show noticeable cytotoxicity except pure DTX, approving the biocompatibility of the fabricated particles (Fig. 6c). Previously, Kusdemir et al. introduced an anti-PSMA antibody conjugated niosome for PSMA-positive cancer treatment [45]. Additionally, Chen et al. optimized an anti-PSMA RNA aptamer-coated liposome against prostate cancer cells [46]. However, our designed nanostructure was more efficient than theirs due to its pH sensitivity.

#### 3.3.4 Apoptosis induction evaluation

**Fig. 7:**
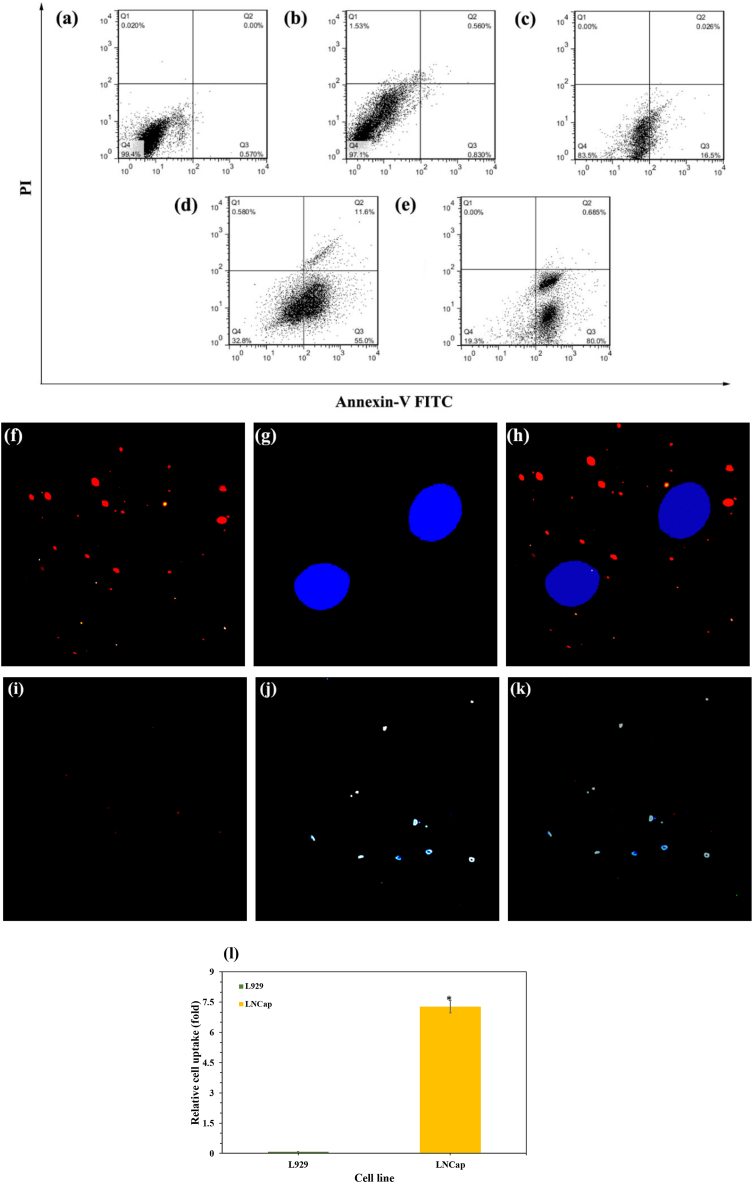
Apoptosis assay of LNCap cells after 24 h of incubation with (a) media, (b) empty pHSN, (c) pure DTX, (d) DTX-pHSN, and (e) AptA10-pHSN. Fluorescence images of (f) QD-attached aptamer strands, (g) DAPI-stained nuclei of LNCap cells, and (h) the merged image of f, and g on LNCap cells. Fluorescence images of (i) QD-attached aptamer strands, (j) DAPI-stained nuclei of LNCap cells, and (k) the merged image of j, and l on L929 cells, and (l) relative internalization of QDs-Apt A10-pHSNs to the LNCap and L929 cell lines.

Annexin-FITC and PI double stainings were used to investigate the types of cell death following exposure to different forms of the treatment. As is clear, the flowcytometric quantifications revealed approximately 0.57, 1.39, 16.52, 66.6, and 80.68 % of apoptotic populations in LNCap cells treated by the medium (control), empty AptA10-pHSNs, pure DTX, DTX-pHSN, and AptA10-DTX-pHSN, respectively (Fig. 7a–7e). The DTX-loaded pHSNs significantly caused 50 % more apoptosis induction in cells. Additionally, the AptA10-DTX-pHSNs increased this effect by ∼30 % in comparison to the DTX-pHSNs, confirming the MTT results which targeting of the expressed PSMA receptors on the surface of the LNCap cells led to maybe more cellular uptake of the DTX-loaded particles [47]. Consequently, more cellular uptake of DTX-loaded particles increased the amount of DTX molecules inside the cells, which enhanced their cytotoxicity on PSMA-positive prostate cancer cells. Additionally, the release of DTX in the acidic pH of the lysosome was promoted by the destruction of the structure by breakage of amid bond between DSPE and CA [16]. It is noticeable that no apoptosis induction of empty AptA10-pHSNs confirmed the biocompatibility of the fabricated nanocarriers which previously was observed in MTT assessment.

#### 3.3.5 Bioimaging

In the last examination, fluorescence microscopy was performed on treated LNCap cells with AptA10-pHSNs to benefit from the fluorescence feature of CdSe/ZnS QDs for tracing the cellular uptake of the particles by endocytosis. As is obvious in Fig. 7f, the attached QDs to the aptamer strands were observed in a green filter of the microscope. In addition, the DAPI-stained nuclei of the LNCap cells are illustrated in Fig. 7g. After merging the images, the accumulation of the QDs around the nuclei of the cells was observed, confirming the entrance of the AptA10-pHSNs to the cytoplasm of the cells (Fig. 7h) [48]. However, no negligible QD accumulation around L929 cells’ nuclei was observed (Fig. 7i–k). Additionally, the relative internalization of Apt A10-pHSNs to the PSMA-positive LNCap was 90 folds more than to the PSMA-negative L929 cell line (Fig. 7l). Overall, it can be said that this QD-conjugated anti-PSMA aptamer also can be regarded as a specific biosensor for the diagnosis of prostate cancer.

## 4 Conclusion

In this study, we fabricated and developed a nanosized pH-responsive niosomal material that was surface-modified with a CdSe/ZnS labeled anti-PSMA aptamer for specific delivery of DTX to the prostate cancer cells. The performance of targeted particles was assessed using MTT assay, apoptosis assay, and fluorescence microscopy. The aptamer-conjugated particles had significantly more toxigenicity on targeted PSMA-positive cancer cells than the PSMA-negative cell lines. Additionally, the CdSe/ZnS conjugated aptamers provided a specific bioimaging agent for simultaneous detection and imaging of the PSMA-positive prostate cancer cells. The obtained results showed that the encapsulation of toxic chemotherapic agents in this bioconjugate niosomal platform can improve the efficiency of the treatment with fewer side effects. However, it must be noted that this study, focusing on *in vitro* cell line experiments, limits insight into complex *in vivo* dynamics and long-term effects. The scalability and clinical feasibility of the methods are crucial but not fully addressed, potentially impacting translation to practical medical applications.

## Funding

This research received no external funding.

## Data availability statement

The data presented in this study are available on request from the corresponding author.

## CRediT authorship contribution statement

**Negar Nasri:** Writing – original draft, Visualization, Software, Methodology, Investigation, Formal analysis, Data curation, Conceptualization. **Shaghayegh Saharkhiz:** Writing – original draft, Visualization, Methodology, Investigation, Formal analysis, Data curation, Conceptualization. **Ghasem Dini:** Writing – review & editing, Validation, Supervision, Resources, Project administration, Funding acquisition, Conceptualization. **Fariba Ghasemvand:** Writing – original draft, Validation, Software, Methodology, Investigation, Formal analysis.

## Declaration of competing interest

The authors declare that they have no known competing financial interests or personal relationships that could have appeared to influence the work reported in this paper.
